# Estimates of Total Burned Surface Area by Emergency Department Clinicians and Burn Specialists

**DOI:** 10.7759/cureus.9362

**Published:** 2020-07-23

**Authors:** Barry Hahn, Seungwhan Alex Roh, Christopher Price, Wayne Fu, Jaclyn Dibello, Nicole Berwald, Josh Greenstein, Jerel Chacko

**Affiliations:** 1 Emergency Medicine, Staten Island University Hospital, Staten Island, USA

**Keywords:** burn, emergency department, body surface area

## Abstract

Introduction

Correctly assessing burn size is extremely important since it is directly associated with a patient’s subsequent management. Further, an accurate assessment of the total body surface area (TBSA) involved is crucial to decide if specialty care in a burn unit is necessary, whereby overestimation has the potential to lead to unnecessary patient transfers and undesirable burdens on the healthcare system and inconvenience to patients. The goal of this study was to identify whether burn injury estimates of TBSA percentage correlate between emergency department (ED) clinician and burn specialists.

Methods

This was a retrospective study conducted between February 1, 2018 and July 31, 2019 of patients with a burn injury who were evaluated by both an ED clinician and a burn specialist during the same ED visit. Charts were reviewed to identify the documentation of TBSA by pre-hospital personnel, ED nursing staff, ED mid-level providers (MLP), ED attending physicians, burn consultant MLPs, and burn consultant attending physicians.

Results

During the study period, 189 subjects with both an ED and burn consultant. The median age was 11 years [interquartile range (IQR) 1-49], and 103 (54%) were males. More than half of the subjects (n=106, 56%) were under the age of 18. There was a statistically significant correlation between estimates of TBSA between ED and burn consultants overall (p<0.0001). Furthermore, there was a statistically significant correlation between ED and burn MLPs (p<0.0001) as well as ED and burn attending physicians (p<0.0001). When adjusted for MLP and attending sex, there was still a correlation among all groups (p<0.0001).

Conclusions

In this study, there was a statistically significant correlation between estimates of TBSA between ED and burn consultants

## Introduction

A combination of the burn mechanism, burn depth, extent, and anatomic location helps determine the overall severity of the burn injury (minor, moderate, severe), which provides general guidance for the preferred disposition and care of these patients. The American Burn Association has used these parameters to establish guidelines for the classification of burn severity. Cutaneous burns are classified according to the depth of tissue injury. The depth of the burn largely determines the healing potential and the need for surgical grafting. The percentage of the total body surface area (TBSA) injured is a crucial independent indicator in treatment planning, particularly in estimating fluid replacement requirements, knowledge of the wound size is essential. The extent of the burn usually described as the percentage of TBSA involved and the depth of the burn described as superficial (first degree), partial (second degree), or full-thickness (third degree). A thorough and accurate estimation of burn size is also essential to guide therapy and to determine when to transfer a patient to a burn center. The extent of the burn injury is expressed as a percentage of the patient's TBSA [1]. Burn size can be estimated in a number of ways [2].

Previous studies have evaluated the assessment of TBSA assessments. Specifically, pediatric patients from referral centers have been compared to those later determined at the receiving burn centers; they found a significant difference in TBSA estimates [3]. Some studies found that the referring institutions overestimated the size of the burn by up to 44% TBSA and that burns between 10% and 19.9% TBSA were overestimated most significantly and most frequently [4,5]. Another study found that there are significant inaccuracies between referring hospital estimated and actual TBSA, which consistently and grossly skew toward overestimation. Further, it was found that the overestimation was associated with inefficiencies in burn care, over-resuscitation, and unnecessary painful procedures, including IV cannulation [6-8]. They also concluded that the overestimation of TBSA was associated with potentially unnecessary transfers, which had implications for both the children and their families. These transfers burden the healthcare system, encumber individual’s families, and in the case of children, can result in families being separate and significant financial loss. Similar findings in adult patients have been presented with an inaccurate ED assessment of TBSA occurring in a third of the population admitted to the burn unit. They found deviations of up to 20% between the ED providers and burn specialist’s assessment, also showing the discrepancy and knowledge deficit [9].

The reasons for TBSA estimation discrepancies remain multifactorial, and variation is seen between burn [10] and non-burn specialists [11]. Few, if any, studies have looked at the estimation of TBSA between ED providers and burn specialists. In this study, we examined the estimates of TBSA involved in burn patients made by ED clinicians and by burn specialists both located in a regional burn center. The goal was to identify whether burn injury estimates of TBSA percentage correlate between ED and burn specialists.

## Materials and methods

This was a retrospective, single-center study that was conducted between February 1, 2018 and July 31, 2019 of patients with a burn injury who were evaluated by both an ED clinician and a burn consultant during the same ED visit. The time frame for this study was used because the current ED electronic medical record also began at that start date and allowed for a comprehensive electronic database. Staten Island University Hospital is a 700-bed, tertiary-care teaching hospital and regional burn center in Staten Island, NY. The ED has an annual census of 95,000 patient visits per year. The hospital's regional burn center is accredited by the department of health as one of four in the New York City region. The local institutional review board approved this study.

All subjects who presented to the ED with the complaint of a burn injury and were also evaluated by a burn consultant were included in the study. Patients with incomplete data were excluded from this study. Four study staff were trained in the study protocol and data abstraction. A pre-designed, standardized case report form was utilized. A fifth study staff member verified the accuracy of data input for a small subset of subjects to eliminate errors and ensure consistency and accuracy. Charts were reviewed to identify the documentation of TBSA by pre-hospital personnel, ED nursing staff, ED mid-level providers (MLP), ED attending physicians, burn consultant MLP and burn consultant attending physicians. MLPs at our institution consist of both resident physicians and physician assistants. Patient demographics that were collected included age, gender, ethnicity, insurance status, comorbidities, time and mechanism of burn, TBSA affected and burn severity.

Data collection and processing

The data were collected and managed using Research Electronic Data Capture (REDCap, Nashville, TN), a secure, web-based application designed to support data capture for research studies. The data were analyzed using descriptive statistical methods and were expressed as frequency counts, and percentages for categorical variables or as mean and standard deviation or median and interquartile range (IQR), as appropriate, for continuous variables. Results were presented as proportions or mean difference, with 95% confidence intervals. The data were tested for normality; since the dataset was not normal, the decision was made to use the non-parametric Kendall’s tau test to measure the ranked association between the ED and the burn group’s assessments. Data analyses were conducted using the Analyse-it version 4.95.4 (Analyse-it Software, Leeds, UK).

## Results

During the study, 876 subjects visited the ED for the chief complaint of a burn. Of these, 189 subjects with both an ED provider and burn consultant, were enrolled in the study. All 189 subjects were included in the final analysis. The median age was 11 years (IQR 1-49), and 103 (54%) were males. More than half of the subjects (n=106, 56%) were under the age of 18. Full demographic data for these subjects can be seen in Table 1.

**Table 1:** Demographics Characteristics of Subjects (N=189)

Table 1. Demographics Characteristics of Subjects (N=189)											
	Composite			Age							
	(n=189)			0-18 (n=106)			19-64 (N=60)			> 65 (n=23)	
Age [median, IQR]	11	(1-49)		2	(1-4)		45	(34-35)		73	(70-79)
Sex (n,%)											
male	103	54%		61	58%		29	27%		13	12%
female	86	46%		45	42%		31	29%		10	9%
Ethnicity (n,%)											
White	94	50%		53	50%		28	26%		13	12%
Black	30	16%		15	14%		13	12%		2	2%
Asian	19	10%		11	10%		5	5%		3	3%
Hispanic	2	1%		1	1%		0	0%		1	1%
Other	44	23%		26	25%		14	13%		4	4%
Insurance Status (n,%)											
Public	117	62%		68	64%		30	28%		19	18%
Private	67	35%		37	35%		26	25%		4	4%
None	5	3%		1	1%		4	4%		0	0%
				106			60			23	
Past Medical History											
None	123			91			30			2	
Circulatory System	37			4			14			19	
Endocrine, Nutritional And Metabolic System	16			0			6			10	
Nervous System	10			3			6			1	
Mental Diseases and Disorders	9			4			3			2	
Respiratory System	7			1			4			2	
Digestive System	9			1			4			4	
Alcohol/Drug Use or Induced Mental Disorders	3			0			3			0	
Kidney And Urinary Tract	6			0			3			3	
Skin, Subcutaneous Tissue And Breast	4			2			1			1	
Musculoskeletal System And Connective Tissue	3			0			1			2	
Blood and Blood Forming Organs and Immunological Disorders	2			0			1			1	
Eye	1			0			0			1	
Male Reproductive System	1			1			0			0	
Infectious and Parasitic DDs	1			0			1			0	

Burns were more likely to occur at home (88%) than any other location and the mode of arrival was commonly via ambulance (77%). Most patients presented to the ED for evaluation within 24 hours of the injury (85%). The most common anatomical location of the burn was the front torso (30%). Table 2 fully describes the etiology and characteristics of burn injuries identified in the study subjects and also stratifies the etiology and characteristics by age.

**Table 2:** Etiology, Characteristics and ED Treatment of Burn Injuries ED: emergency department

Table 2. Etiology, Characteristics and ED Treatment of Burn Injuries											
	Composite			Age							
Location (n, %)	(n=189)			0-18 (n=106)			19-64 (N=60)			> 65 (n=23)	
Home	167	88%		94	89%		50	83%		23	100%
Work	7	4%		1	1%		6	10%		0	0%
School	1	1%		1	1%		0	0%		0	0%
Other	14	7%		10	9%		4	7%		0	0%
Mode of Arrival (n, %)											
Ambulance	146	77%		82	77%		44	73%		20	87%
Private Car	35	19%		21	20%		12	20%		2	9%
n/a	8	4%		3	3%		4	7%		1	4%
Time of Occurrence (n, %)											
< 24 h	160	85%		98	92%		45	75%		17	74%
24-48h	18	9%		7	7%		8	13%		3	13%
>48h	9	5%		1	1%		6	10%		2	9%
n/a	2	1%		0	0%		1	2%		1	4%
Mechanism (n, %)											
Scalding	137	72%		92	87%		36	60%		9	39%
Flame	18	10%		1	1%		9	15%		8	35%
Contact	26	14%		11	10%		9	15%		6	26%
Chemical	5	3%		1	1%		4	7%		0	0%
Electrical	1	1%		0	0%		1	2%		0	0%
Other	2	1%		1	1%		1	2%		0	0%
Anatomical Location (n, %)											
Torso Front	57	30%		38	36%		15	25%		4	17%
Lower Extremity	55	29%		30	28%		14	23%		11	48%
Upper Extremity	42	22%		20	19%		19	32%		3	13%
Head / Face	21	11%		10	9%		8	13%		3	13%
Torso Back	11	6%		8	8%		2	3%		1	4%
Other	3	2%		0	0%		2	3%		1	4%
ED Treatment (n,%)											
Antibiotics	112	59%		62	33%		38	20%		12	6%
Analgesics / Narcotic	109	58%		61	32%		37	20%		11	6%
Analgesics / Non-narcotic	27	14%		20	11%		6	3%		1	1%
Mechanical Ventilation	3	2%		0	0%		1	1%		2	1%
Other	1	1%		0	0%		0	0%		1	1%
Disposition (n, %)											
Admit ICU	119	63%		63	59%		40	67%		16	70%
Admit Floor	34	18%		19	18%		10	17%		5	22%
Admit Observation	1	1%		0	0%		1	2%		0	0%
Admit Telemetry	1	1%		0	0%		1	2%		0	0%
Discharge	34	18%		24	23%		8	13%		2	9%

Of the 189 subjects, 144 (76%) had an ED MLP and ED attending, as well as a burn MLP and burn attending whom each documented the burn TBSA. There were 45 (24%) subjects with either an ED MLP or ED attending as well as a burn MLP or burn attending who documented the burn injury TBSA. Only 35 (19%) subjects had a pre-hospital provider and 53 (28%) ED nurses document the burn injury TBSA.

As shown in Table 3, there was a statistically significant correlation between estimates of TBSA between ED and burn consultants overall (p<0.0001). Furthermore, there was a statistically significant correlation between ED and burn MLPs (p<0.0001) or ED and burn attending physicians (p<0.0001). When adjusted for MLP and attending sex, there was still a correlation among any of the groups (p<0.0001). Table 4 stratifies the correlation of TBSA estimate by age group.

**Table 3:** Correlation Between Documented TBSA TBSA: total body surface area

Table 3. Correlation Between Documented TBSA								
		ALL		Males		Females		P-value
Mid-Level Provider		N=130		N=70		N=60		
correlation coefficient		0.719		0.715		0.692		<0.00001
Attending		N=110		N=60		N=50		
correlation coefficient		0.709		0.666		0.714		<0.00001
Overall		N=189		N=103		N=86		
correlation coefficient		0.686		0.665		0.665		<0.00001

**Table 4:** Correlation Between Documented TBSA Stratified by Age TBSA: total body surface area

Table 4. Correlation Between Documented TBSA Stratified by Age								
		0-18 years		19-64 years		65+		P-value
Mid-Level Provider vs Mid-Level Provider		N=74		N=38		N=18		
correlation coefficient		0.619		0.851		0.727		<0.001
Attending vs Attending		N=60		N=39		N=11		
correlation coefficient		0.703		0.697		0.762		<0.001
Emergency Department Providers vs Burn Specialist		N=106		N=60		N=23		
correlation coefficient		0.645		0.739		0.699		<0.001

## Discussion

The percentage of TBSA injured is an essential indicator of severity. In treatment planning, particularly in estimating fluid replacement requirements, knowledge of the wound size is essential. It is well known among burn physicians that referring providers from outside hospitals often misestimate the TBSA of burns [12]. Few, if any, studies have assessed burn size estimation within the same institution. The goal of the current study was to identify whether burn injury estimates of TBSA percentage correlate between ED and burn specialists at a regional burn center. We found a statistically significant correlation between estimates of TBSA between ED and burn consultants overall (p<0.0001). This study did not attempt to identify the reasons as to why there was a correlation between ED and burn providers. Anecdotally, there is no single method of TBSA that ED providers used. There are many possibilities for this finding, and future studies should focus on which TBSA estimation methods and communication processes facilitated this finding. Prior studies did identify a potential difference in TBSA estimate between sexes [13]. It is possible that the findings were different in this study as compared to previous studies done by burn specialists due to research bias or other factors. We found no difference between estimates of TBSA between ED and burn consultants across both patient sex and age.

The median age of subjects in this study was 11 years, and more than half of the subjects were under the age of 18. These demographics mirrors previous studies, which included all age groups and were conducted in other countries [14-16]. We also found that 54% of subjects were male. Variances among males and females for burn incidence vary by age, region, and income [17]. Some theories for these differences include the tendency for males to more frequently engage in dangerous behaviors and that men may work in more injury-prone environments [18].

Similar to data published by the American Burn Association [19], in this study, we found that burns were more likely to occur at home (88%). We found that the most common anatomical location of the burn was the front torso (30%). Other studies have cited various locations as the most common site of injury. Different explanations have been offered as to why different anatomical locations may be more commonly affected [20-22]. For instance, one paper stated that since individuals tend to use their hands as a reflex to protect themselves, the hands, arms, face, and legs are more commonly injured [23]. In the end, it is clear that different burns, as well as their anatomical location, are related to the geographic location [8].

This was a retrospective chart review. Therefore, subject to the limitations of such studies. Furthermore, the study was conducted at a single center, which was also a regional burn center. Therefore, although many of the findings in this study were similar to those in other studies, the generalization of the results may still be limited.

Due to the retrospective nature of this study, we were unable to identify the method that clinicians used to measure TBSA. Several methods are routinely employed to estimate the TBSA affected. More common methods include; (1) Rule of Nines, where a percentage that is either nine or a multiple of nine to determine how much body surface area is damaged, (2) Lund and Browder Chart which considers the age of the person, with decreasing TBSA for the head and increasing percentage of body surface area (BSA) for the legs as the child ages, making it more useful in pediatric burns and (3) Palmar Method, which equates the patient’s palmar surface to 1% TBSA. Computerized methods of TBSA estimation, including smartphone apps, are also available but have not yet been widely accepted. It is unclear if the same or varying methods were used. Future studies should evaluate whether there is a more consistent method of TBSA assessment.

## Conclusions

There was a high degree of correlation between burn injury estimates of TBSA between ED providers and burn specialists at a single regional, northeast burn center. Further studies may be necessary to confirm these findings on a larger scale and among other clinicians. This study did not attempt to identify the reasons as to why there was a correlation between ED and burn providers. Anecdotally, there is no single method of TBSA that ED providers used. There are many possibilities for this finding, and future studies should focus on which TBSA estimation methods and communication processes facilitated this finding.
